# Large language model–based prediction of speech intelligibility after Vibrant Soundbridge implantation using multidimensional outcome data: Part 2 of a prospective study

**DOI:** 10.1038/s41598-025-20919-5

**Published:** 2025-11-12

**Authors:** Christoph Müller, Hannes Seidler, Anna Tsypina, Janina Kuch, Thomas Zahnert, Susen Lailach

**Affiliations:** 1 Department of Otorhinolaryngology, Head and Neck Surgery, Ear Research Center Dresden, TU Dresden, Medical Faculty and University Hospital Carl Gustav Carus, Dresden, Germany; 2 Carl Gustav Carus Faculty of Medicine, Technische Universität Dresden, Dresden, Germany

**Keywords:** Active middle ear implant, Vibrant soundbridge, Large language model, Prediction, Outcome parameter, Speech intelligibility, Sensory systems, Outcomes research, Translational research, Data processing

## Abstract

Active middle ear implants (AMEIs) such as the Vibrant Soundbridge (VSB) offer an effective treatment option for patients with mixed or conductive hearing loss and large air–bone gaps, where conventional hearing rehabilitation often fails. However, postoperative outcomes—particularly speech intelligibility at 65 dB SPL in free-field (WRS_65dB_)—show high interindividual variability. This study aimed to develop a predictive model for WRS_65dB_ based on four clinically relevant parameters: postoperative bone conduction thresholds (BC_PTA4_), unaided preoperative maximum speech intelligibility (WRS_max_), Vibrogram threshold (VIB_PTA4_), and age. Data from 20 patients were analyzed. Spearman’s correlation revealed significant associations between WRS_65dB_ and postoperative BC_PTA4_, preoperative WRS_max_, and age. Using a seven-step approach supported by GPT-4o, we developed a sigmoid-transformed linear regression model. The final model included BC_PTA4_, WRS_max_, and age and achieved an R² of 0.51, *r* = 0.71, RMSE = 6.18, and MAE = 4.67. Model performance was assessed by means of residual and outlier analysis. This model provides a transparent and clinically applicable tool for preoperative outcome estimation in VSB candidates. Further validation in larger, multicenter cohorts is needed to confirm its generalizability.

**Supplementary Information:**

The online version contains supplementary material available at 10.1038/s41598-025-20919-5.

## Introduction

The success of conventional hearing rehabilitation in patients with mixed (MHL) or conductive (CHL) hearing loss (middle ear surgery followed by postoperative fitting of conventional hearing aids) is often limited in cases involving large air-bone gaps. Large air-bone gaps require high amplification power and output level, which conventional hearing aids frequently cannot provide. In such cases, active middle ear implants (AMEIs), such as the Vibrant Soundbridge (VSB, MED-EL, Innsbruck, Austria) - currently the only AMEI commercially available - offer a reliable alternative.

Despite its clinical value, outcomes after VSB implantation vary substantially between individuals, highlighting the need for personalized prognostic assessment. A key outcome parameter is postoperative speech intelligibility as measured by the Freiburg monosyllabic word test in free-field at 65 dB (WRS_65dB_).

Cochlear capacity is a major determinant of postoperative speech intelligibility^1,2^. The most commonly used parameter for its assessment is the pure-tone average (PTA) of bone conduction (BC) thresholds at 0.5, 1, 2, and 4 kHz (BC_PTA4_). Evidence suggests that manufacturer-recommended BC_PTA4_ thresholds overestimate the optimal indication range. Considering device limitations such as maximum power output and a limited dynamic range (30–35 dB), a BC_PTA4_ of 47 dB to 52 dB appears to represent a realistic upper limit for favorable postoperative outcomes after VSB implantation^3^. We presented data^4^ with BC_PTA4_ thresholds within this restricted indication range, suggesting an adequate cochlear reserve across the cohort. Nevertheless, considerable variability in postoperative speech intelligibility was observed, with WRS_65dB_ values ranging from 60% to 95% (Δ = 35%). The averaged BC_PTA4_ threshold showed a strong and statistically significant Spearman’s correlation (ρ = −0.522, *p* = 0.018) with the postoperative WRS_65dB_. By contrast, a previous study^2^ found no such association, likely due to the inclusion criterion of preoperative unaided maximum speech intelligibility (WRS_max_) ≥ 95%, which reduced the variability required to detect a correlation between WRS_65dB_ and BC_PTA4_. WRS_max_ represents an alternative parameter for characterizing cochlear reserve and predicting postoperative outcome in both conventional hearing aid and cochlear implant users^5,6^. In our data^4^, WRS_max_ ranged from 60% to 100% and showed a high and significant Spearman’s correlation (ρ = 0.555, *p* = 0.011) with WRS_65dB_, confirming its predictive value in the context of vibroplasty.

Coupling quality referred to as coupling efficiency (CE_PTA4_) represents another factor influencing WRS_65dB_. It is defined as the difference between the in situ thresholds Vibrogram (VIB_PTA4_) and BC_PTA4_. In our data^4^ Spearman’s correlation indicated an inverse relationship (ρ = −0.136, *p* = 0.569) between CE and WRS_65dB_. A strong negative correlation (ρ = −0.464, *p* = 0.040) was found between VIB_PTA4_ and WRS_65dB_. A coupling loss of less than 20 dB is generally associated with satisfactory speech understanding, typically around 75%^2,4^. Nevertheless, WRS_65dB_ can still vary considerably despite similar coupling efficiency. One explanation is the influence of the coupling technique and the coupler type. Round window (RW) coupling is known to yield lower coupling efficiency—particularly in the low-frequency range—when compared to ossicular chain coupling^7^. CE data must be interpreted with caution. The reference values for Vibrogram measurements are derived from temporal bone studies using coupling of the FMT to the long process of the incus with an expected variability of ± 10 dB^8^. Precise calibration data for other coupling methods remain unavailable. Under optimal conditions—i.e., ideal coupling to the long incus process—a difference of 0 dB across all frequencies would be expected, indicating perfect mechanical energy transfer. Positive values indicate an unfavorable coupling.

Effective gain (EG_PTA4_) is defined as the difference between free-field thresholds (FF_PTA4_) and BC_PTA4_. It quantifies the extent to which cochlear hearing loss can be compensated by the implant^9^. Unexpectedly, our recent data showed a positive Spearman’s correlation between EG_PTA4_ and WRS_65dB_, indicating improved speech intelligibility with increasing EG. This finding contradicts the expected inverse relationship. One possible explanation lies in the limitations of aided thresholds measurements used to calculate EG. Intrinsic microphone noise, compression and feedback suppression algorithms, as well as individual patient tolerance may all compromise threshold accuracy and thus affect calculation of EG values. In addition, EG data reflect mechanical transmission characteristics of the VSB with relatively high (poorer) EG values at low frequencies and progressively lower (better) EG values toward the mid- and high-frequencies^1,7,10^. Furthermore, EG is influenced by the type of vibroplasty. RW coupling is associated with poorer EG values, particularly at low frequencies, compared to ossicular chain coupling.

Beyond identifying individual influencing factors on audiological outcomes, their systematic evaluation is essential for developing predictive scoring systems. Among the influencing factors discussed above, recent data^4^ identified three independent audiological parameters of postoperative aided WRS_65dB_: postoperative BC_PTA4_, unaided WRS_max_ and VIB_PTA4_. In addition, ‘age’ was found to be a significant negative predictor.

This manuscript—part two of a two-part publication series—presents a predictive model developed with the support of the Large Language Model GPT-4o. The model estimates postoperative speech intelligibility (WRS_65dB_) following VSB implantation based on the four parameters: postoperative BC_PTA4_, unaided preoperative WRS_max_, VIB_PTA4_, and age.

## Results

Data of 20 patients (sex: 10 female and 10 male patients) were evaluated. **Tab.** **1** summarizes demographic details, etiology of hearing loss, coupling site and coupler type. In 8 patients, the cause of hearing loss was chronic otitis media without cholesteatoma (COMsC), in 11 patients it was chronic otitis media with cholesteatoma (COMcC). In 13 of 20 patients, the round window was chosen as the coupling site. 10 of these 13 patients were implanted with a round window soft coupler (RWSC), 3 received a round window titanium coupler (RWTC). **Tab.** **2** presents the statistical analysis of the four parameters evaluated in our cohort for inclusion in the predictive model of WRS_65dB_: postoperative BC_PTA4_, unaided preoperative WRS_max_, VIB_PTA4_, and age. Please refer to **Supp. 1**, **Tab.** **A1**, **A2** and **Fig.** **A1** for detailed analyses of the processed raw data and corresponding graphical visualization. For further statistical analyses and interpretations of the data from our patient cohort, please refer to Müller et al.^4^.

**Table 1:** Details regarding the implanted patients. * The time of the calculation is referenced to the date of the study measurements. COMsC (chronic otitis media without cholesteatoma), COMcC (chronic otitis media with cholesteatoma), round window (RW), oval window (OW), SP (short process), RWTC (round window titanium coupler), RWSC (round window soft coupler), SClip (stapes clip coupler), SSH (stapes SH coupler), OWC (oval window coupler), SP (incus short process coupler).

Item Patient	Age	Time since VSB implantation in months*	Etiology of hearing loss	Coupling site	Coupler
ID					
1 (mild outlier in BC_PTA4_)	78	6	COMsC, multiple ear surgeries	RW	RWTC
2	58	12	COMsC, tympanosclerosis	RW	RWSC
3	30	11	COMcC, multiple ear surgeries, radical cavity, external ear canal stenosis	RW	RWSC
4 (mild outlier in CE_PTA4_)	36	7	external ear canal atresia, stapes fixation	RW	RWSC
5	67	11	COMsC, multiple ear surgeries, tympanosclerosis	RW	RWTC
6	57	40	COMcC, multiple ear surgeries, radical cavity	RW	RWSC
7	35	23	COMsC, condition after TBC with labyrinthitis	OW	OWC
8	41	46	COMcC, multiple ear surgeries, radical cavity	RW	RWSC
9	33	15	COMsC, multiple ear surgeries	Stapes	SClip
10	61	49	COMcC, multiple ear surgeries, radical cavity	Stapes	SClip
11	32	43	COMsC, condition after stapes surgery in external hospital	RW	RWSC
12	27	25	COMcC, multiple ear surgeries, radical cavity, condition after flooting footplate	RW	RWSC
13	30	105	COMcC, multiple ear surgeries, change from BB to VSB due to recurrent headaches	RW	RWSC
14	46	37	COMcC, multiple ear surgeries, wet radical cavity	OW	OWC
15	69	48	COMcC, multiple ear surgeries, radical cavity, post-inflammatory stapes fixation	RW	RWSC
16	54	107	COMcC, multiple ear surgeries, radical cavity	RW	RWSC
17	37	3	COMcC, multiple ear surgeries, radical cavity, external ear canal stenosis	Stapes OW	SSH
18 (strong outlier in EG_PTA4_)	28	3	COMcC, multiple ear surgeries, radical cavity	OW	OWC
19	53	3	COMsC, condition after release of malleus head fixation	Incus SP	SP
20 (low performer)	68	5	COMsC, tympanosclerosis, post-inflammatory stapes fixation	RW	RWTC

**Table 2:** Summary of statistical analysis of the four parameters evaluated in our cohort for inclusion in the predictive model. Bone conduction (BC_PTA4_), maximum unaided preoperative speech intelligibility in the Freiburg monosyllabic word test at 65 dB SPL in free-field (WRS_max_), vibrogram (VIB_PTA4_), age, postoperative aided speech intelligibility in the Freiburg monosyllabic word test at 65 dB SPL in free-field (WRS_65dB_).

	BC_PTA4_	WRS_max_	VIB_PTA4_	age	WRS_65dB_
Mean ± SD	28.0 ± 10.1 dB	88.5 ± 12.3%	40.2 ± 11.9 dB	47.0 ± 16.1 y	81.5 ± 9.0%
Range (minimum…maximum)	3.75…50 dB	60…100%	16.3…61.3 dB	27…78 y	60…95% (low performer ID 20)

### Sigmoid-transformed linear regression model

**Figure 1:**
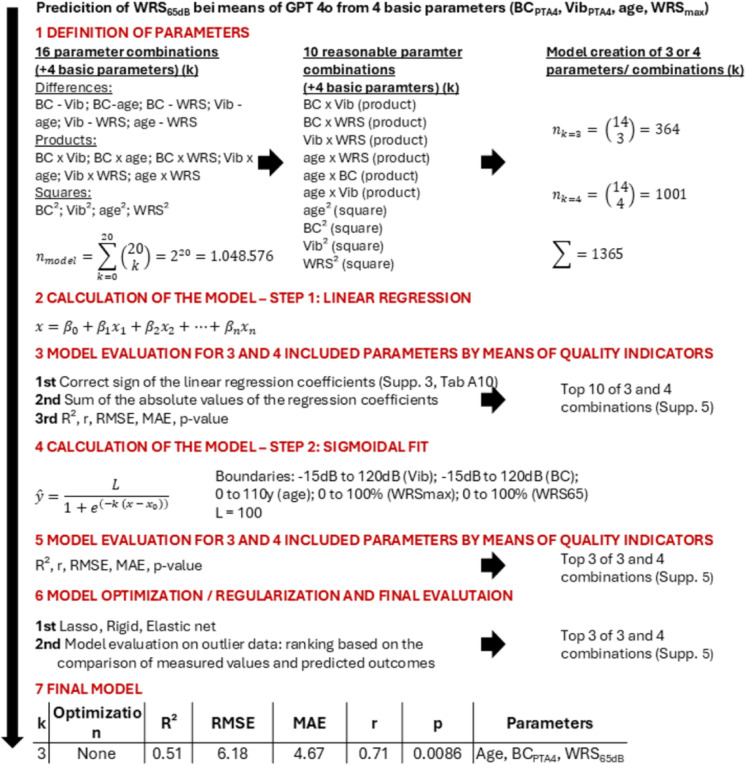
Workflow for the computation of the sigmoid-transformed linear regression model in seven steps.

**Figure** **1** illustrates the workflow for the computation of the sigmoid-transformed linear regression model, as described in the Methods section. Ultimately, only three parameters (age, WRS_max_ and BC_PTA4_) were included in the model, while VIB_PTA4_ was excluded during step 3 of the calculation process. The outcome of the linear regression is presented in **Eq**. **1**, and the sigmoid fit is provided in **Eq**. **2**. The statistical analysis of the linear regression is summarized in **Tab**. **3**. Only the intercept (β₀) and the parameter “age” reached statistical significance in the linear regression.

**Eq**. **1** Linear regression of estimated postoperative speech intelligibility.

1 $$\:{WRS}_{65dB}=\:\frac{100}{1+\:{e}^{(-0.063*(x-57.2941)}}\:\:$$

**Table 3:** Linear regression coefficients, standard error, t-value, 95% confidence interval and p-values of the linear regression. Statistical significance was found only for the intercept (β_0_) and the parameter ‘age’. * statistically significant.

	Regression coefficient	Standard error	T-value	95% confidence interval	*P*-value
β_0_	79.614	16.651	4.781	[44.314; 114.913]	0.000204*
β_1_	−0.2809	0.120	−2.341	[−0.535; −0.026]	0.0325*
β_2_	−0.0795	0.184	−0.431	[−0.470; 0.311]	0.672
β_3_	0.1957	0.147	1.334	[−0.115; 0.507]	0.201

The goodness of fit of the sigmoid-transformed linear regression model was evaluated using standard metrics (**Tab.** **4**). The three-parameter model achieved an R² value of 0.51, indicating that approximately 51% of the variance in postoperative aided WRS_65dB_ could be explained by the model, corresponding to a moderate model fit. The RMSE was 6.18, and the MAE was 4.67, reflecting a moderate average deviation between predicted and observed values. The correlation coefficient (r) between the predicted and observed outcomes was 0.71, indicating a substantial positive relationship (moderate correlation). Overall, the model demonstrated statistical significance (F(3,16) = 5.51, *p* = 0.0086), underscoring that the observed associations are unlikely to be attributable to random variation. Although only the parameter “age” achieved statistical significance at the individual predictor level, the model as a whole exhibited a statistically significant fit after sigmoid transformation.

**Eq.** **2** Sigmoid fit of linear regression of estimated postoperative speech intelligibility (F(3, 16) = 5.51, p = 0.0086).

2 $$\:x={{\upbeta\:}}_{0}+{{\upbeta\:}}_{1}*age+{{\upbeta\:}}_{2}*{BC}_{PTA}+{{\upbeta\:}}_{3}*\:{WRS}_{max}$$

**Table 4:** Quality of the sigmoid fit of the linear regression model: R² (coefficient of determination), RMSE (root mean squared error), MAE (mean absolute error) and r (correlation coefficient). No optimization (e.g. LASSO, rigid, elastic net) was used for the final model built.

K	Optimization	*R*²	RMSE	MAE	*R*	*P*-value	Parameters
3	None	0.51	6.18	4.67	0.71	0.0086	Age, BC_PTA4_, WRS_max_

**Figure 2:**
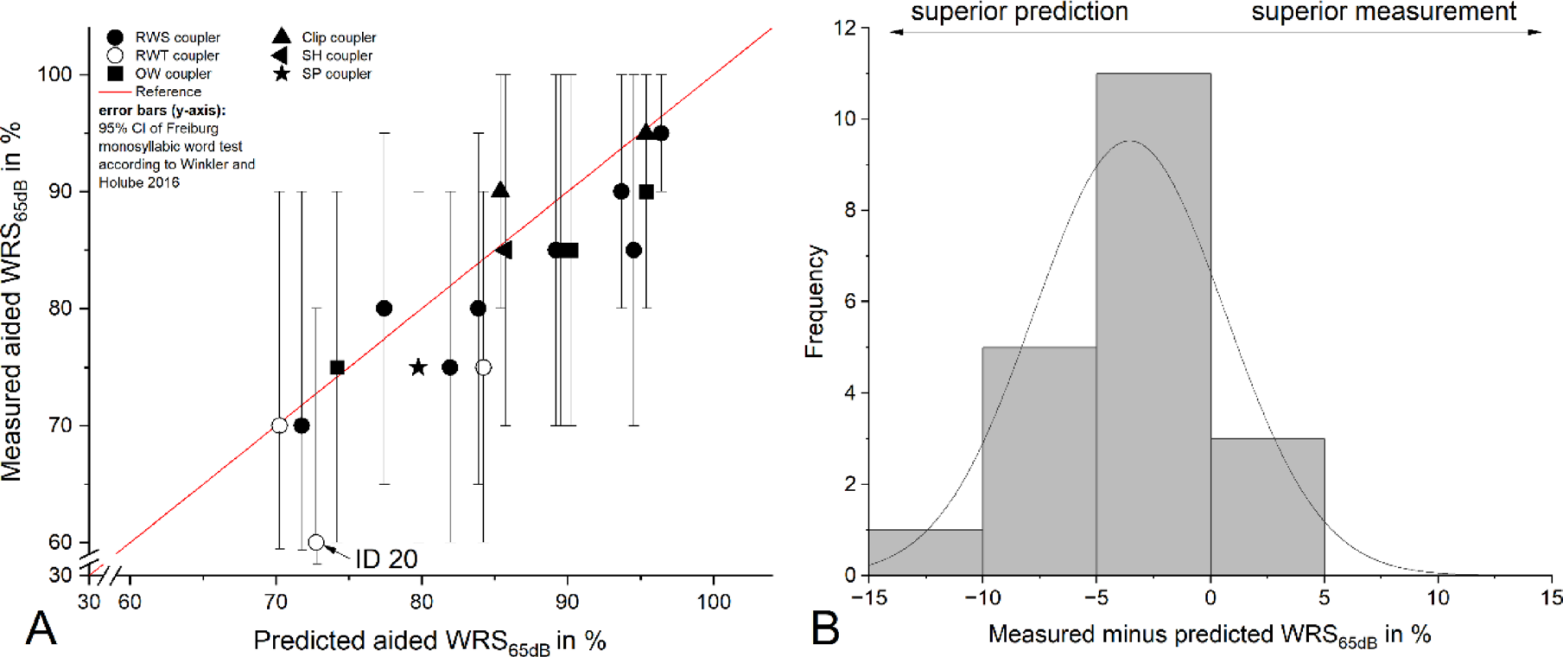
(**A**) relationship between the predicted aided WRS_65dB_ and the corresponding measured values after adjusting the measurements according to the 95% confidence interval of the Freiburg monosyllabic speech test as described by Winkler and Holube 2016. IDs 7, 10 and 15 demonstrate better-than-predicted outcome. (**B**) Difference between measured data and predictions: the histogram of the residuals demonstrates an approximately normal distribution. ID 20 could be defined as a statistical outlier.

The experimental data were correlated with the model prediction after the completion of the model calculation (**Fig**. 2A). The measurements were corrected for the 95% confidence interval of the Freiburg monosyllabic word test (Winkler and Holube, 2016). All IDs fell within the 95% confidence interval of the prediction. IDs 7 (OW coupler), 10 (SClip coupler), and 15 (RW soft coupler) performed above the prediction, although the differences did not reach statistical significance. The measurements of all other IDs were below the prediction, likewise without statistical significance. Analysis of the residuals (difference between the measured and predicted values of aided WRS_65dB_) revealed an approximately normal distribution of the measurements within the prediction (**Fig**. 2B). The first quartile (Q1) of the residuals was − 5.26, the third quartile (Q3) was − 0.65, resulting in an interquartile range (IQR) of 4.60. Only low performer ID 20 lay outside the interval [Q1/Q3 ± 1.5×IQR], with a residual of − 12.75, and could therefore be defined as a statistical outlier.

## Discussion

This study presents a predictive model for postoperative speech intelligibility (WRS_65dB_) after VSB implantation, developed with the support of the Large Language Model GPT-4o. Four parameters were considered: postoperative BC_PTA4_, unaided WRS_max_, VIB_PTA4_ and age. The final model included three parameters: BC_PTA4_, unaided WRS_max_ and age.

### Positioning the speech intelligibility prediction model in clinical and scientific contexts

In recent years, numerous studies have developed predictive models to estimate postoperative speech intelligibility following the implantation of active hearing systems. These approaches differ in terms of outcome variables, model complexity, predictor variables, and validation strategies.

The most methodologically robust models to date were published by Hoppe et al.^5,11^. In a retrospective study of 128 CI patients with moderate to severe hearing loss (≤ 80 dB HL) and preserved residual hearing, Hoppe et al.^5^ showed by means of a generalized linear regression model that maximum unaided speech intelligibility (WRS_max_), WRS_65dB_ with conventional hearing aid (WRS_65dB HA_) and age significantly correlate with postoperative WRS_65dB_ with CI (WRS_65dB CI_). In 98% of cases, the postoperative WRS_65dB CI_ 6 month after surgery exceeded the WRS_max_ of at least 15% (median WRS_65dB CI_ = 70%), making the latter a conservative predictor of minimum expected outcome. 75% of included CI recipients reached a score not poorer than 12% below the predicted WRS_65dB CI_. Further model development^11^ was performed from data of 165 postlingually deafened patients by including patients with residual (*n* = 109, group 1, WRS_max_ > 0%) and no residual (*n* = 56, group 2, WRS_max_ = 0%) preoperative speech intelligibility. The three standardized preoperative audiometric parameters from earlier studies^5^— WRS_65 HA_, WRS_max_ and age – were adopted with their regression coefficients kept fixed. In addition, the model was expanded by one parameter: duration of unaided hearing impairment (DuHI) or duration of hearing impairment (DHI) in case that DuHI could not be obtained. When both parameters were available, only DuHI was used, as DHI did not show a significant effect in the presence of DuHI. For patients with WRS_max_ > 0%, predictive accuracy – measured by means of the MAE – remained nearly stable (11.1%) compared to the former study^5^. For patients with WRS_max_ = 0%, MAE was significantly reduced to 17.0% compared to the former study (23.7%).

For Vibrant Soundbridge users, Koyama et al.^12^ applied both multivariate linear regression and a machine learning approach (random forest model) to predict postoperative WRS based on data of 30 patients. The following parameters - age at implantation, FMT location, coupler usage, sex, AC and BC thresholds, as well as FF thresholds with and without CHA - were used as predictors for speech intelligibility in quiet 3 month postoperatively. The multiple linear regression analysis yielded an R² of 0.59 and a MAE of 0.12, while the random forest model achieved an R² of 0.85 and an MAE of 0.06. In the linear model, WRS with CHA, age, and coupler usage emerged as the most influential predictors, whereas in the random forest model, WRS with CHA was the dominant predictor. Unfortunately, the authors did not provide their models, preventing external validation.

In the domain of transcutaneous bone conduction systems, Wimmer et al.^13^ demonstrated that BC_PTA4_ of the better-hearing ear is the strongest predictor of postoperative WRS_65dB_ with Bonebridge in 30 patients (31 implantations) with MHL, CHL or single sided deafness (SSD) by means of a logistic regression model. BC_PTA4_ values ≤ 38 dB HL contributed in 95% of the patients to a postoperative WRS_65dB_ ≥ 75%. No significant effect of the poorer ear, language, or indication group (CHL/MHL vs. SSD) was found. Unfortunately, the authors didn’t report model quality parameters (e.g., R², MAE).

Another logistic regression model was developed by Arndt et al.^14^ for 29 patients (36 implantations) with CHL (CHL group: BC_PTA4_ < 20 dB) or MHL (MHL I group: BC_PTA4_ > 20 dB and MHL II group: 20 dB < BC_PTA4_ < 40 dB) receiving the Osia bone conduction system to analyze the prediction of postoperative WRS_65dB_ based on preoperative unaided WRS_max_. The logistic regression analysis yielded an R² of 0.72 (CHL group) and 0.61 (MHL I group). No prediction of MHL II group was possible because of a wide range of the results.

Taken together, all studies demonstrate that relatively simple models based on a small number of audiological predictors—especially WRS_max_, BC_PTA4_, age, and in some cases WRS_65dB HA_—can predict postoperative speech understanding with moderate to high accuracy. The present study introduced a sigmoid-transformed linear regression model to predict postoperative speech intelligibility (WRS_65dB_) after VSB implantation, based on a systematic 7-step model-building approach. Four core predictors were selected based on Spearman correlations and clinical plausibility: age, BC_PTA4_, VIB_PTA4_, and WRS_max_. Second-degree polynomial terms and interaction effects were systematically explored across all 3- and 4-parameter combinations (*n* = 1365 models). The final model (age, BC_PTA4_, WRS_max_) achieved an R² of 0.51, MAE of 4.67, RMSE of 6.18, and a Pearson correlation (r) of 0.71, indicating a moderate-to-strong model fit. Unlike prior studies, the model included automated regularization (LASSO, Ridge, Elastic Net) to prevent overfitting. It further incorporates a sign-constrained coefficient plausibility check, ensuring that all selected models are clinically interpretable. Moreover, by correcting calculated WRS_65dB_ scores using the 95% confidence interval of the Freiburg monosyllabic word test (**Fig**. 2B), the model demonstrates methodological rigor and practical interpretability.

### Limitations

Despite its clinical relevance and robust statistical performance, the proposed predictive model is subject to limitations that must be considered when interpreting the results. The model was developed based on data from a single center with a limited number of patients. This may reduce the generalizability of the findings and increase the risk of overfitting, particularly for more complex predictor combinations. Due to the limited number of patients receiving this type of implant per year, we did not have an additional test dataset beyond the training dataset used. Consequently, our model has not been validated against an independent dataset, which represents a major limitation of the study.

The model focuses on audiological parameters and patient age. It does not account for other intraoperative factors (e.g. coupling site and couplers, implant positioning), which are known to influence audiological outcomes and may introduce unmeasured confounding. While the type of coupler used is likely to influence audiological outcomes^2,8^ and may represent a clinically meaningful predictor, its inclusion in the present model was not statistically justified due to the limited subgroup sizes. The small number of cases per coupler type would have resulted in low statistical power and an increased risk of overfitting.

The calculations are based on postoperative BC thresholds. Although no significant differences were found between preoperative (data not shown) and postoperative BC thresholds, we chose to use the postoperative values to strengthen the methodological robustness of this study. Under the assumption that BC thresholds remain stable postoperatively, using preoperative BC thresholds in the predictive model would have emphasized its predictive character.

Speech intelligibility is a complex process influenced by central auditory processing. One potentially relevant factor is the duration of inadequately compensated hearing loss. Patients with mixed or conductive hearing loss frequently undergo multiple ear surgeries and experience recurrent otorrhea, during which hearing aids cannot be worn. Evidence from cochlear implant research^11^ suggests that this parameter may significantly contribute to the variability of auditory outcomes.

Future studies should focus on multicenter validation in larger and more heterogeneous populations, include model stratification by coupler type, duration of hearing loss, and cognitive screening scores, systematically compare the performance of different machine learning approaches, and validate the models using independent datasets.

## Conclusion

To predict postoperative speech intelligibility, we developed a sigmoid-transformed linear regression model based on age, BC_PTA4_ and unaided WRS_max_. The final model achieved good predictive accuracy (R² = 0.51, *r* = 0.71, RMSE = 6.18, MAE = 4.67) and was validated using residual and outlier analysis. The model offers a transparent and clinically applicable tool to support preoperative counseling and indication decision-making in VSB candidates.

## Materials and methods

This single-center study was conducted between March 1, 2023, and February 28, 2024, at a tertiary referral center (Department of Otorhinolaryngology, Head and Neck Surgery, University Hospital Carl Gustav Carus, Technische Universität Dresden). The study was carried out in accordance with the Declaration of Helsinki and the principles of good clinical practice, following approval by the local ethics committee of Technische Universität Dresden (BO-EK-51012023).

The study follows a two-step publication approach for presenting the data. Part 1^4^ focused on the comprehensive statistical analysis of outcome parameters, whereas this paper – part 2 – builds on those data to develop a predictive model of postoperative speech intelligibility.

### Inclusion criteria

Adult VSB users with acquired hearing loss who had undergone unilateral implantation at the study site were included in the analysis. In all cases, the bone conduction (BC) and air conduction (AC) thresholds on the contralateral (non-implanted) side—measured at 0.5, 1, 2, and 4 kHz and calculated as the pure-tone average (PTA4)—did not exceed 30 dB, indicating that no hearing device was required on that side. All patients were implanted in accordance with international consensus guidelines^9^. Please refer to Müller et al. 2025 regarding the surgical procedure of the VSB implantation.

Demographic details on the patients included, information on the implantation period, coupling site, applied coupler and etiology of hearing loss are provided in **Tab.** **1**.

### Exclusion criteria

Patients were excluded from the study if they were under 18 years of age, unable to provide informed consent, unable to comply with study instructions, or diagnosed with neurological or psychological disorders.

### Timing of data collection

All measurements were conducted once during the study period. At the time of testing, all patients had received their initial fitting at least three months earlier.

### Initial and follow-up fitting

Initial fitting was performed six weeks after implantation using the DSL [I/O] fitting formula, based on Vibrogram thresholds. A follow-up fitting was conducted four weeks later, taking into account patient feedback and audiometric results.

### Measurements

Details of the acquired audiological data are provided in Müller et al. 2025 ^4^, the following section provides a summary.

### Audiological measurements

The audiometric measurements were conducted in a soundproof room (DIN EN ISO 8253) by means of a clinical audiometer (AT900 or AT1000, Auritec GmbH, Hamburg, Germany). The processed raw data from the audiological measurements are available in **Supp. **1, **Tab**. **A1** and **A2**.

### Threshold-based measurements

At the implanted side, postoperative BC thresholds between 0.125 and 6 kHz were measured using pure-tone audiometry with BC headphones (KLH 96, CB-Elmec, Germany). FF thresholds were assessed using narrowband noise with center frequencies ranging from 0.25 to 8 kHz. To ensure adequate masking, the contralateral ear was occluded using a conventional earplug (SNR = 27 dB) and additionally covered with earmuffs (Peltor Optime III H540A, SNR = 35 dB, 3 M, Minnesota, USA). The most recent air conduction (AC) thresholds, obtained prior to implantation (0.125–8 kHz), were retrieved from the patient records stored in the clinical information system at the study site. The Vibrogram (VIB) was obtained by delivering the stimulus directly to the FMT. Hearing thresholds were determined using the Hughson–Westlake method and expressed in decibels. The following outcome parameters were calculated from the threshold-based measurements described above:

For BC, AC, FF thresholds and VIB, the PTA was calculated across the frequencies 0.5, 1, 2, and 4 kHz (PTA4) and 1, 2, and 4 kHz (PTA3), respectively. The effective gain (EG, PTA4 or PTA3) was calculated as the difference between the FF threshold and the postoperative BC. The coupling efficiency (CE, PTA4 or PTA3) was calculated as the difference between the Vibrogram threshold and the postoperative BC. The dynamic range (DR, PTA4 or PTA3) was calculated as the difference between the aided FF threshold and the maximum power output of the VSB (75 dB (0.5 kHz), 83 dB(1.0 kHz), 90 dB (2 kHz), 80 dB (4 kHz)^3^.

### Speech intelligibility testing

Aided speech intelligibility was assessed using the Freiburg monosyllabic word test in quiet at 65 dB SPL in the free field (WRS_65dB_), with the contralateral ear plugged and covered as previously described. Speech was presented towards the front of the patient’s head (0°). The most recent preoperative maximum unaided speech intelligibility (WRS_max_) was retrieved from the clinical records stored in the hospital’s information system. It had been assessed using the Freiburg monosyllabic word test in quiet, with sound presented via headphones and the contralateral ear masked using variable-level speech noise.

### Calculations and statistical analyses of audiological measurements

All calculations and statistical analyses of the audiological data were performed by means of Microsoft Excel (Microsoft Corporation, Redmont, U.S.), Origin (OriginLab, Northampton, U.S.) and SPSS (IBM, Armonk, U.S.). All statistical tests were performed at the significance level of α = 0.05. When the text refers to a trend or tendency, the results are not significant (*p* ≥ 0.05).

Müller et al. 2025^4^ have already conducted a detailed statistical analysis of the audiological data used in this manuscript (threshold-based audiometry and speech audiometry) by means of dispersion measures (mean, median, 25% (Q1) to 75% (Q3) percentile, lower whisker (Q1 −1.5x interquartile range (IQR)), upper whisker (Q3 + 1.5x IQR)). ANOVAs with post hoc analyses were performed to evaluate differences in distribution across frequencies, as well as between PTA3 and PTA4. The corresponding graphical representation can be found in **Supp**. **1**, **Fig.** **A1**.

Monotonic correlations between aided WRS_65dB_ and the aforementioned threshold-based parameters, as well as patient age and unaided WRS_max_, were analyzed using Spearman’s correlation coefficient (ρ) (**Supp**. 2, **Tab.** **A3**). Among the threshold-based parameters, PTA4 consistently yielded stronger correlation coefficients than PTA3. Linear regression lines were fitted (**Supp. 2**, **Fig. ****A2**,** Tab. ****A4**). Correlation strength was interpreted according to Cohen’s guidelines: low/weak (|ρ or R| = 0.10), moderate (|ρ or R| = 0.30), and high/strong (|ρ or R| = 0.50) (Cohen, 2007). An aided WRS_65dB_ score of < 70% was defined as the threshold distinguishing high performers (HP; WRS ≥ 70%) from low performers (LP; WRS < 70%). This cut-off was based on multicenter clinical data reporting a mean aided WRS_65dB_ of 72% for the current (third) generation of couplers^15^. In our dataset, only one patient (ID 20) fell into the low-performer category. Residual analysis was conducted to identify potential outliers.

Based on the correlation coefficients and p-values (**Supp. 2**, **Tab. ****A3**), the following four parameters were selected for further calculations in the prediction model of WRS_65dB_: age, WRS_max_, BC_PTA4_, and VIB_PTA4_.

### Prediction model based on sigmoid fit of a linear regression with second-degree polynomial terms

Model fitting and statistical calculations were performed within the embedded Python environment of ChatGPT-4o (OpenAI, version available on March 31, 2025), using standard scientific libraries (e.g., scikit-learn, statsmodels). Results were obtained interactively through code-based computation and validated by the authors. Please refer to **Fig.** **1** to overview our 7-step approach of model building.

During step 1 Spearman’s correlation coefficients (**Supp. 2**, **Tab.** **A3** and **A4**, **Fig. ****A2**) were applied to identify four basic outcome parameters—age, BC_PTA4_, VIB_PTA4_, and WRS_max_—for inclusion in the regression model to predict aided WRS_65dB_ as the main outcome parameter. To account for potential non-linear relationships, all parameters were z-standardized, and linear, quadratic, and interaction terms (second-degree polynomials) were included in the model (**Fig.** **1**, 1 st step, left side and **Supp. 3**, **Tab**. **A5**). In addition to the 4 basic parameters and based on clinical relevance, 10 quadratic terms or combinations of different parameters were identified as potential contributors to the linear regression (**Fig.** **1**, 1st step, middle and **Supp. 4**, **Tab**. **A6**** to** **A8**). Based on the available dataset, preliminary calculations (not shown) indicated that for combinations of up to a maximum of four different parameters, all signs of the regression coefficients in the linear regression models corresponded to those of the calculated correlations between parameter and WRS_65dB_ (**Supp. 3**, **Tab**. **A5**).

During step 2, linear regression models were computed for all possible combinations of three (n = 364) and four (n = 1001) out of the 14 selected parameters, comprising 4 basic parameters, 4 quadratic terms, and 6 interaction terms.

During step 3, the ten best models for each set of three- or four-parameter combinations were identified based on the correctness of the regression coefficient signs, the sum of the absolute values of the regression coefficients within the model, and standard regression metrics (**Supp. 6**) (coefficient of determination (R²), Pearson’s correlation coefficient (r), root mean square error (RMSE), mean absolute error (MAE), and p-value **(Supp. 5**, **Tab**. **A9**** to** **A12**). At this stage, the final selection of three parameters—BC_PTA4_, WRS_max_ and age—was made, while VIB_PTA4_ was excluded from the model.

During step 4, the sigmoid fit was calculated for each of the ten best-performing linear regression models with three or four combined predictors, based on predefined boundaries for the parameters VIB_PTA4_, BC_PTA4_, WRS_max_, and WRS_65dB_.

During step 5, model quality was assessed using the above-mentioned standard regression metrics.

(R², r, RMSE, MAE) to select the three best models for each set of 3 and 4 parameters (**Supp. 5**, **Tab**.** A13**).

During step 6, several regularization strategies were applied. LASSO (Least Absolute Shrinkage and Selection Operator) eliminates unnecessary parameters by setting some coefficients to zero. Ridge regression shrinks coefficients toward zero without eliminating any predictors. Elastic Net combines the properties of both LASSO and Ridge regression (**Supp. 5**, **Tab**. **A14**). All models were then applied to the initial dataset, and the accuracy of predictions for the four identified outliers (IDs 6, 15, 17, and 19 — defined as cases where the difference between the observed and predicted value exceeded the model-specific MAE) was evaluated (**Supp**. **5**, **Tab**. **A15**). Models were ranked based on the absolute prediction error for these outliers (with smaller differences indicating better performance). The final model, which achieved the lowest average rank (**Supp**. **5**, **Tab**. **A16**) in this outlier prediction task, was selected based on a comprehensive evaluation of all metrics, including R², Pearson’s r, MAE, RMSE, the sum of absolute coefficients, and average rank in outlier prediction .

### Evaluating prediction model against our own data

The prediction model results were compared to our measured data of the aided WRS_65dB_ (**Fig**. 2**A**), which were corrected for the 95% confidence interval of the test-retest reliability of the Freiburg monosyllabic word test^16^. The differences between our measured vs. predicted data of the aided WRS_65dB_ were in addition plotted as a histogram (**Fig.** 2**B**). Outliers in the residuals were identified using the interquartile range (IQR) method. Residuals outside the intervals [Q1 − 1.5×IQR] and [Q3 + 1.5×IQR] were classified as outliers.

## Supplementary Information

Below is the link to the electronic supplementary material.

Supplementary Material 1

Supplementary Material 2

Supplementary Material 3

Supplementary Material 4

Supplementary Material 5

## Data Availability

The processed raw data of all patients/subjects are attached in the appendix.

## Abbreviations

AC: Air conduction
AMEI: Active middle ear implant
BC: Bone conduction
CE: Coupling efficiency
CHA: Conventional hearing aid
CHL: Condurctive hearing loss
COMcC: Chronic otitis media with cholesteatoma
COMsC: Chronic otitis media without cholesteatoma
DSL [i/o]: Desired sensation level input/output
DR: Dynamic range
EG: Effective gain
FF: Free-field
FMT: Floating mass transducer
IQR: Interquartile range
LP: Long process of the incus
LLM: Large Language Model
MAE: Mean absolute error
MHL: Mixed hearing loss
OC: Ossicular chain
PTA: Pure tone average
ρ: Spearman’s correlation coefficient
RW: Round window
RWSC/RWS coupler: Round window soft coupler
RWTC/RWT coupler: Round window titanium coupler
SClip/SClip coupler: Stapes Clip coupler
SD: Standard deviation
SH: Stapes head
SSH/SH cupler: Stapes SH coupler
SP: Short process of the incus
SPC: / SP coupler short process coupler
SPL: Sound pressure level
VIB: Vibrogram
VSB: Vibrant Soundbridge
WRS: Word recognition score
WRS_65dB_: Aided speech intelligibility at 65 dB SPL
WRS_max_: Maximum unaided speech intelligibility

## Declarations

### Use of translation aids

During manuscript preparation, DeepL and GPT-4o were utilized to translate the manuscript into English.
