# Supplementary material for: Large language model–based prediction of speech intelligibility after Vibrant Soundbridge implantation using multidimensional outcome data: Part 2 of a prospective study
Source: Sci Rep. 2025 Nov 12;15:39564. doi: 10.1038/s41598-025-20919-5 (PMC12612111; doi:10.1038/s41598-025-20919-5)
Supplement: Supplementary file 1 — Supplementary Material 1 [file 41598_2025_20919_MOESM1_ESM.pdf]

Supplemental File 1 – Processed audiological raw data

ID	Pat.	Age	Bone conduction (BC) in dB HL						Vibrogram (Vib) in dB						Free Field (FF) threshold in dB HL						WRS 65dB aided in %	WRS max unaided in %
			0,5 kHz	1,0 kHz	2,0 kHz	4,0 kHz	PTA4	PTA3	0,5 kHz	1,0 kHz	2,0 kHz	4,0 kHz	PTA4	PTA3	0,5 kHz	1,0 kHz	2,0 kHz	4,0 kHz	PTA4	PTA3		
1	VSB30	78	15	50	65	70	50,0	61,7	65	55	55	70	61,3	60,0	35	40	40	60	43,8	46,7	70	70
2	VSB04	58	20	30	30	30	27,5	30,0	55	45	30	35	41,3	36,7	45	40	35	30	37,5	35,0	70	60
3	VSB03	30	15	25	20	25	21,3	23,3	45	30	15	20	27,5	21,7	30	30	20	30	27,5	26,7	90	100
4	VSB35	36	10	20	20	25	18,8	21,7	70	70	35	45	55,0	50,0	25	25	40	35	31,3	33,3	85	70
5	VSB23	67	25	45	45	35	37,5	41,7	25	40	35	55	38,8	43,3	30	35	25	35	31,3	31,7	75	80
6	VSB01	57	30	35	35	30	32,5	33,3	45	45	30	25	36,3	33,3	30	35	35	30	32,5	33,3	80	90
7	VSB06	35	20	25	35	55	33,8	38,3	40	20	20	45	31,3	28,3	30	30	35	55	37,5	40,0	75	80
8	VSB10	41	20	35	45	30	32,5	36,7	40	30	25	25	30,0	26,7	30	25	25	30	27,5	26,7	95	100
9	VSB17	33	15	20	25	20	20,0	21,7	45	30	25	20	30,0	25,0	40	35	50	40	41,3	41,7	95	100
10	VSB12	61	5	15	35	40	23,8	30,0	45	40	25	40	37,5	35,0	30	25	20	35	27,5	26,7	90	85
11	VSB15	32	25	30	25	15	23,8	23,3	40	30	25	25	30,0	26,7	35	35	25	40	33,8	33,3	85	100
12	VSB05	27	0	5	25	45	18,8	25,0	45	30	35	60	42,5	41,7	15	15	35	65	32,5	38,3	90	90
13	VSB13	30	45	45	50	30	42,5	41,7	65	65	45	40	53,8	50,0	55	50	50	30	46,3	43,3	85	100
14	VSB02	46	20	20	25	40	26,3	28,3	30	25	25	65	36,3	38,3	40	25	30	50	36,3	35,0	85	95
15	VSB09	69	5	25	40	30	25,0	31,7	55	45	45	55	50,0	48,3	35	30	35	50	37,5	38,3	80	95
16	VSB14	54	10	15	30	30	21,3	25,0	40	30	30	30	32,5	30,0	35	30	30	30	31,3	30,0	75	100
17	VSB39	37	5	20	40	65	32,5	41,7	35	35	40	70	45,0	48,3	35	35	50	50	42,5	45,0	85	90
18	VSB40	28	5	5	5	0	3,8	3,3	35	15	10	5	16,3	10,0	40	35	55	50	45,0	46,7	85	100
19	VSB41	53	25	35	35	50	36,3	40,0	55	60	60	70	61,3	63,3	30	30	35	50	36,3	38,3	75	75
20	VSB33	68	25	35	30	40	32,5	35,0	55	45	40	50	47,5	45,0	35	35	35	45	37,5	38,3	60	90

Tab A1 Age and Hearing results I (raw data): bone conduction, vibrogram, free field threshold, preoperative unaided maximum speech intelligibility (WRSmax) measured with headphones, postoperative aided speech intelligibility at 65dB (WRS65) measured in free field conditions.

ID	Pat.	Coupling Efficiency (Vib - BC) in dB						Effective Gain (FF - BC) in dB						VSB output in dB HL				Dynamic Range (VSB output - FF) in dB					
		0,5 kHz	1,0 kHz	2,0 kHz	4,0 kHz	PTA4	PTA3	0,5 kHz	1,0 kHz	2,0 kHz	4,0 kHz	PTA4	PTA3	0,5 kHz	1,0 kHz	2,0 kHz	4,0 kHz	0,5 kHz	1,0 kHz	2,0 kHz	4,0 kHz	PTA4	PTA3
1	VSB30	50	5	-10	0	11,3	-1,7	20	-10	-25	-10	-6,3	-15,0	75	83	90	80	40	43	50	40	43,3	44,3
2	VSB04	35	15	0	5	13,8	6,7	25	10	5	0	10,0	5,0	75	83	90	80	30	43	55	45	43,3	47,7
3	VSB03	30	5	-5	-5	6,3	-1,7	15	5	0	5	6,3	3,3	75	83	90	80	45	53	70	60	57,0	61,0
4	VSB35	60	50	15	20	36,3	28,3	15	5	20	10	12,5	11,7	75	83	90	80	50	58	50	40	49,5	49,3
5	VSB23	0	-5	-10	20	1,3	1,7	5	-10	-20	0	-6,3	-10,0	75	83	90	80	45	48	65	55	53,3	56,0
6	VSB01	15	10	-5	-5	3,8	0,0	0	0	0	0	0,0	0,0	75	83	90	80	45	48	55	45	48,3	49,3
7	VSB06	20	-5	-15	-10	-2,5	-10,0	10	5	0	0	3,8	1,7	75	83	90	80	45	53	55	45	49,5	51,0
8	VSB10	20	-5	-20	-5	-2,5	-10,0	10	-10	-20	0	-5,0	-10,0	75	83	90	80	45	58	65	55	55,8	59,3
9	VSB17	30	10	0	0	10,0	3,3	25	15	25	20	21,3	20,0	75	83	90	80	35	48	40	30	38,3	39,3
10	VSB12	40	25	-10	0	13,8	5,0	25	10	-15	-5	3,8	-3,3	75	83	90	80	45	58	70	60	58,3	62,7
11	VSB15	15	0	0	10	6,3	3,3	10	5	0	25	10,0	10,0	75	83	90	80	40	48	65	55	52,0	56,0
12	VSB05	45	25	10	15	23,8	16,7	15	10	10	20	13,8	13,3	75	83	90	80	60	68	55	45	57,0	56,0
13	VSB13	20	20	-5	10	11,3	8,3	10	5	0	0	3,8	1,7	75	83	90	80	20	33	40	30	30,8	34,3
14	VSB02	10	5	0	25	10,0	10,0	20	5	5	10	10,0	6,7	75	83	90	80	35	58	60	50	50,8	56,0
15	VSB09	50	20	5	25	25,0	16,7	30	5	-5	20	12,5	6,7	75	83	90	80	40	53	55	45	48,3	51,0
16	VSB14	25	15	0	0	10,0	5,0	25	15	0	0	10,0	5,0	75	83	90	80	40	53	60	50	50,8	54,3
17	VSB39	30	15	0	5	12,5	6,7	30	15	10	-15	10,0	3,3	75	83	90	80	40	48	40	30	39,5	39,3
18	VSB40	30	10	5	5	12,5	6,7	35	30	50	50	41,3	43,3	75	83	90	80	35	48	35	25	35,8	36,0
19	VSB41	30	25	25	20	25,0	23,3	5	-5	0	0	0,0	-1,7	75	83	90	80	45	53	55	45	49,5	51,0
20	VSB33	30	10	10	10	15,0	10,0	10	0	5	5	5,0	3,3	75	83	90	80	40	48	55	45	47,0	49,3

Tab A2 Hearing results II (raw data): coupling efficiency, effective gain, VSB maximum output hearing level (Rahne & Plontke 2022), dynamic range (Rahne et al. 2016).

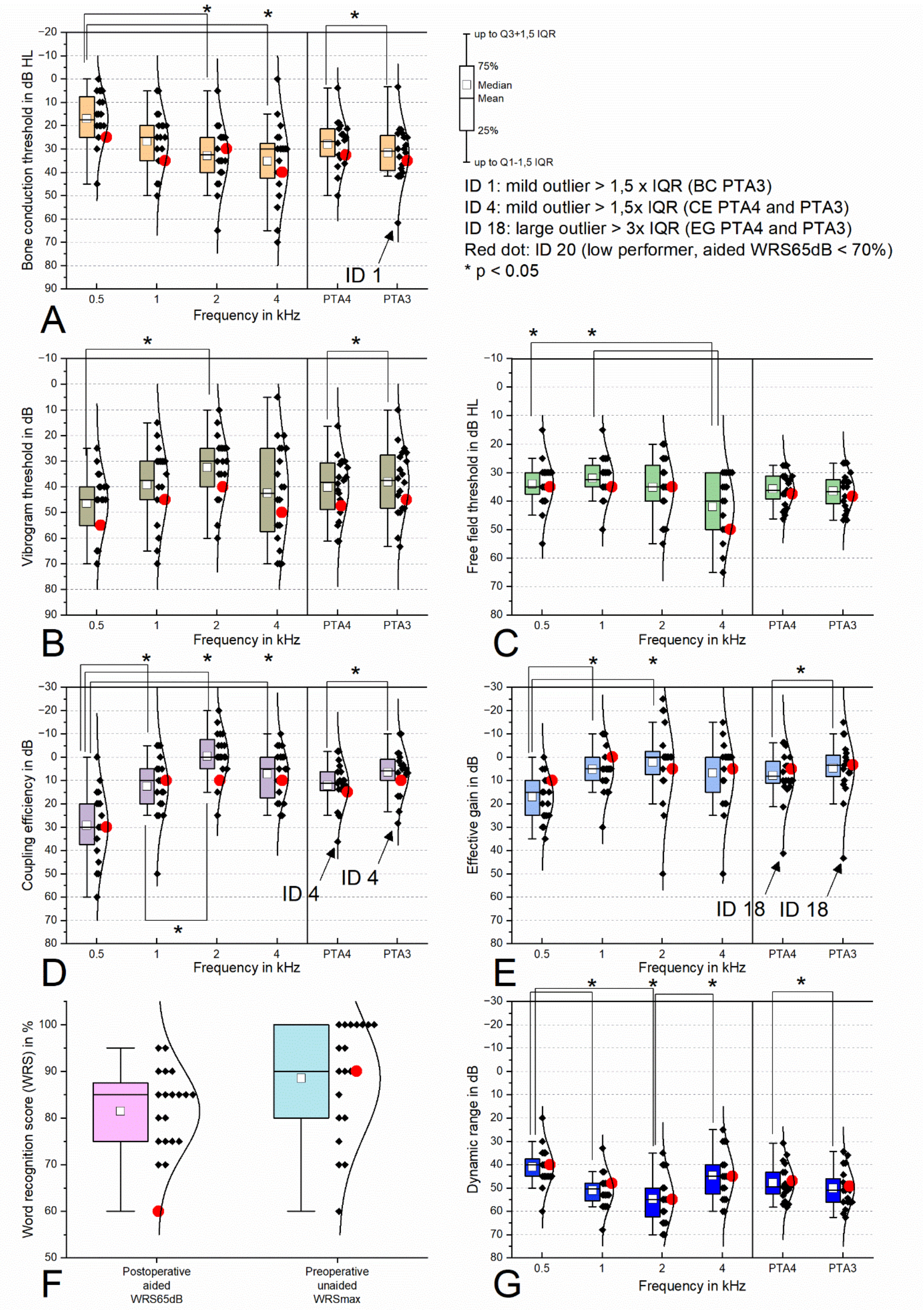

Fig A1 Visual representation of threshold-based parameters (A–E, G) and postoperative speech intelligibility (F).
