# Supplementary material for: Large language model–based prediction of speech intelligibility after Vibrant Soundbridge implantation using multidimensional outcome data: Part 2 of a prospective study
Source: Sci Rep. 2025 Nov 12;15:39564. doi: 10.1038/s41598-025-20919-5 (PMC12612111; doi:10.1038/s41598-025-20919-5)
Supplement: Supplementary file 2 — Supplementary Material 2 [file 41598_2025_20919_MOESM2_ESM.pdf]

Supplemental file 2 – Spearman Correlation Coefficient (non-parametric testing) including p-Values for prediction parameters

		Age	BC PTA4	BC PTA3	Vib PTA4	Vib PTA3	FF PTA4	FF PTA3	WRS65	WRSmax	CE PTA4	CE PTA3	EG PTA4	EG PTA3	EG PTA4 exID18	EG PTA3 exID18	Dynamic Range PTA4	Dynamic Range PTA3	Difference WRSmax WRS65
Age	Correlation coeff.	1,000	.476*	.487*	.454*	0,436	-0,066	-0,146	-.626**	-.499*	0,131	-0,028	-.476*	-.532*	-0,392	-.458*	-0,005	0,069	-0,074
	Sig. (2-sided)		0,034	0,029	0,044	0,054	0,782	0,538	0,003	0,025	0,583	0,906	0,034	0,016	0,097	0,049	0,984	0,773	0,755
	N	20	20	20	20	20	20	20	20	20	20	20	20	20	19	19	20	20	20
BC PTA4	Correlation coeff.	.476*	1,000	.947**	.460*	.517*	0,258	0,196	-.522*	-0,360	-0,268	-0,267	-.837**	-.815**	-.809**	-.784**	-0,203	-0,153	-0,033
	Sig. (2-sided)	0,034		0,000	0,041	0,020	0,271	0,408	0,018	0,118	0,253	0,256	0,000	0,000	0,000	0,000	0,390	0,519	0,889
	N	20	20	20	20	20	20	20	20	20	20	20	20	20	19	19	20	20	20
BC PTA3	Correlation coeff.	.487*	.947**	1,000	.547*	.601**	0,239	0,231	-.457*	-0,391	-0,142	-0,187	-.775**	-.814**	-.737**	-.783**	-0,160	-0,135	-0,098
	Sig. (2-sided)	0,029	0,000		0,013	0,005	0,311	0,327	0,043	0,088	0,549	0,430	0,000	0,000	0,000	0,000	0,501	0,571	0,682
	N	20	20	20	20	20	20	20	20	20	20	20	20	20	19	19	20	20	20
Vib PTA4	Correlation coeff.	.454*	.460*	.547*	1,000	.983**	0,261	0,314	-.464*	-.633**	.656**	.574**	-0,233	-0,279	-0,103	-0,158	-0,245	-0,301	-0,329
	Sig. (2-sided)	0,044	0,041	0,013		0,000	0,267	0,178	0,040	0,003	0,002	0,008	0,324	0,234	0,675	0,519	0,297	0,197	0,157
	N	20	20	20	20	20	20	20	20	20	20	20	20	20	19	19	20	20	20
Vib PTA3	Correlation coeff.	0,436	.517*	.601**	.983**	1,000	0,252	0,306	-.447*	-.593**	.582**	.563**	-0,278	-0,314	-0,156	-0,199	-0,219	-0,266	-0,284
	Sig. (2-sided)	0,054	0,020	0,005	0,000		0,283	0,189	0,048	0,006	0,007	0,010	0,236	0,178	0,525	0,415	0,355	0,257	0,224
	N	20	20	20	20	20	20	20	20	20	20	20	20	20	19	19	20	20	20
FF PTA4	Correlation coeff.	-0,066	0,258	0,239	0,261	0,252	1,000	.945**	-0,301	-0,005	0,208	0,187	0,223	0,212	0,114	0,101	-.899**	-.862**	0,206
	Sig. (2-sided)	0,782	0,271	0,311	0,267	0,283		0,000	0,198	0,984	0,378	0,431	0,344	0,370	0,642	0,680	0,000	0,000	0,383
	N	20	20	20	20	20	20	20	20	20	20	20	20	20	19	19	20	20	20
FF PTA3	Correlation coeff.	-0,146	0,196	0,231	0,314	0,306	.945**	1,000	-0,227	-0,090	0,278	0,225	0,246	0,224	0,134	0,110	-.801**	-.810**	0,068
	Sig. (2-sided)	0,538	0,408	0,327	0,178	0,189	0,000		0,335	0,706	0,235	0,339	0,296	0,343	0,584	0,655	0,000	0,000	0,777
	N	20	20	20	20	20	20	20	20	20	20	20	20	20	19	19	20	20	20
WRS65	Correlation coeff.	-.626**	-.522*	-.457*	-.464*	-.447*	-0,301	-0,227	1,000	.555*	-0,136	-0,085	0,336	0,292	0,332	0,285	0,304	0,285	-0,111
	Sig. (2-sided)	0,003	0,018	0,043	0,040	0,048	0,198	0,335		0,011	0,569	0,723	0,147	0,211	0,165	0,238	0,193	0,224	0,640
	N	20	20	20	20	20	20	20	20	20	20	20	20	20	19	19	20	20	20
WRSmax	Correlation coeff.	-.499*	-0,360	-0,391	-.633**	-.593**	-0,005	-0,090	.555*	1,000	-0,367	-0,185	0,317	0,346	0,246	0,278	0,000	0,079	.719**
	Sig. (2-sided)	0,025	0,118	0,088	0,003	0,006	0,984	0,706	0,011		0,111	0,434	0,173	0,135	0,309	0,248	0,999	0,740	0,000
	N	20	20	20	20	20	20	20	20	20	20	20	20	20	19	19	20	20	20
CE PTA4	Correlation coeff.	0,131	-0,268	-0,142	.656**	.582**	0,208	0,278	-0,136	-0,367	1,000	.870**	0,426	0,337	0,434	0,339	-0,207	-0,258	-0,259
	Sig. (2-sided)	0,583	0,253	0,549	0,002	0,007	0,378	0,235	0,569	0,111		0,000	0,061	0,146	0,064	0,156	0,382	0,271	0,271
	N	20	20	20	20	20	20	20	20	20	20	20	20	20	19	19	20	20	20
CEPTA3	Correlation coeff.	-0,028	-0,267	-0,187	.574**	.563**	0,187	0,225	-0,085	-0,185	.870**	1,000	.491*	.471*	.516*	.492*	-0,176	-0,212	-0,049
	Sig. (2-sided)	0,906	0,256	0,430	0,008	0,010	0,431	0,339	0,723	0,434	0,000		0,028	0,036	0,024	0,032	0,459	0,369	0,837
	N	20	20	20	20	20	20	20	20	20	20	20	20	20	19	19	20	20	20
EG PTA4	Correlation coeff.	-.476*	-.837**	-.775**	-0,233	-0,278	0,223	0,246	0,336	0,317	0,426	.491*	1,000	.970**	1.000**	.965**	-0,214	-0,236	0,134
	Sig. (2-sided)	0,034	0,000	0,000	0,324	0,236	0,344	0,296	0,147	0,173	0,061	0,028		0,000		0,000	0,364	0,316	0,574
	N	20	20	20	20	20	20	20	20	20	20	20	20	20	19	19	20	20	20
EG PTA3	Correlation coeff.	-.532*	-.815**	-.814**	-0,279	-0,314	0,212	0,224	0,292	0,346	0,337	.471*	.970**	1,000	.965**	1.000**	-0,200	-0,223	0,204
	Sig. (2-sided)	0,016	0,000	0,000	0,234	0,178	0,370	0,343	0,211	0,135	0,146	0,036	0,000		0,000		0,397	0,344	0,388
	N	20	20	20	20	20	20	20	20	20	20	20	20	20	19	19	20	20	20
EG PTA4 exID18	Correlation coeff.	-0,392	-.809**	-.737**	-0,103	-0,156	0,114	0,134	0,332	0,246	0,434	.516*	1.000**	.965**	1,000	.965**	-0,105	-0,129	0,039
	Sig. (2-sided)	0,097	0,000	0,000	0,675	0,525	0,642	0,584	0,165	0,309	0,064	0,024		0,000		0,000	0,670	0,598	0,875
	N	19	19	19	19	19	19	19	19	19	19	19	19	19	19	19	19	19	19
EG PTA3 exID18	Correlation coeff.	-.458*	-.784**	-.783**	-0,158	-0,199	0,101	0,110	0,285	0,278	0,339	.492*	.965**	1.000**	.965**	1,000	-0,088	-0,115	0,120
	Sig. (2-sided)	0,049	0,000	0,000	0,519	0,415	0,680	0,655	0,238	0,248	0,156	0,032	0,000		0,000		0,719	0,641	0,626
	N	19	19	19	19	19	19	19	19	19	19	19	19	19	19	19	19	19	19
Dynamic Range PTA4	Correlation coeff.	-0,005	-0,203	-0,160	-0,245	-0,219	-.899**	-.801**	0,304	0,000	-0,207	-0,176	-0,214	-0,200	-0,105	-0,088	1,000	.975**	-0,197
	Sig. (2-sided)	0,984	0,390	0,501	0,297	0,355	0,000	0,000	0,193	0,999	0,382	0,459	0,364	0,397	0,670	0,719		0,000	0,405
	N	20	20	20	20	20	20	20	20	20	20	20	20	20	19	19	20	20	20
Dynamic Range PTA3	Correlation coeff.	0,069	-0,153	-0,135	-0,301	-0,266	-.862**	-.810**	0,285	0,079	-0,258	-0,212	-0,236	-0,223	-0,129	-0,115	.975**	1,000	-0,089
	Sig. (2-sided)	0,773	0,519	0,571	0,197	0,257	0,000	0,000	0,224	0,740	0,271	0,369	0,316	0,344	0,598	0,641	0,000		0,710
	N	20	20	20	20	20	20	20	20	20	20	20	20	20	19	19	20	20	20
Difference of WRSmax and WRS65	Correlation coeff.	-0,074	-0,033	-0,098	-0,329	-0,284	0,206	0,068	-0,111	.719**	-0,259	-0,049	0,134	0,204	0,039	0,120	-0,197	-0,089	1,000
	Sig. (2-sided)	0,755	0,889	0,682	0,157	0,224	0,383	0,777	0,640	0,000	0,271	0,837	0,574	0,388	0,875	0,626	0,405	0,710	
	N	20	20	20	20	20	20	20	20	20	20	20	20	20	19	19	20	20	20

*Tab A3 Spearman correlation coefficients and two-sided testing for significance. * and **: the correlation is significant (* p<0.05 (yellow shaded), ** p< 0.01 (green shaded); both two-sided testing for significance). Correlation coefficients are interpreted according to Cohen: low / weak correlation |ρ or r| = 0.10, medium / moderate correlation |ρ or R| = 0.30, high / strong correlation |ρ or R| = 0.50 (Cohen 2007). The red-shaded parameters are visualized in detail in the manuscript and are partly part of the predictor model. Note that the calculated correlation for the effective gain (EG_PTA4_exID18) does not match with the clinical experience: Higher EG (higher difference between FF and BC) should be accompanied by lower aided WRS65.*

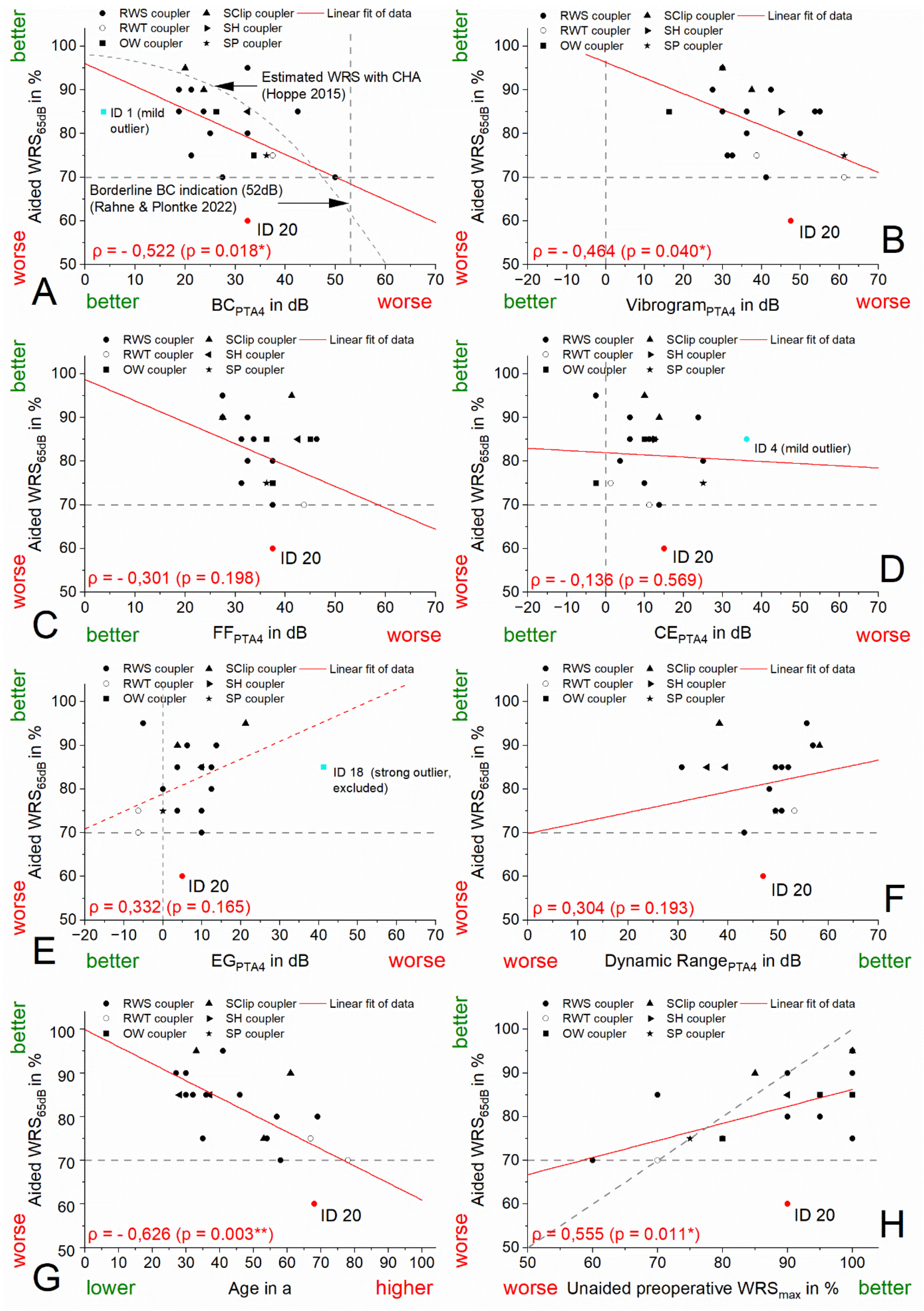

Fig A2 Correlations of threshold based predictive parameters (A: BC_{PTA4}, B: Vib_{PTA4}, C: FF_{PTA4}, D: CE_{PTA4}, E: EG_{PTA4}, F: DR_{PTA4}) and additional non-threshold-based paramters (G: age, H: unaided preoperative WRS_{max}) with postoperative aided speech intelligibility (WRS_{65dB}; dashed line at 70% indicate cut-of between high and low performance). Strong significant correlations ($|\rho| > 0.5$, $p<0.05$) for BC_{PTA4}, age, unaided preoperative WRS_{max}. Moderate significant correlations ($0.3 < |\rho| < 0.5$, $p<0.05$) for Vib. Moderate non-significant correlations ($0.3 < |\rho| < 0.5$, $p>0.05$) for FF, EG, DR. Weak non-significant correlation ($0.1 < |\rho| < 0.3$, $p<0.05$) for CE_{PTA4}. Data of Hoppe et al. 2015 and Rahne and Plontke et al. 2022 are added to Fig A.

Dependent Parameter (y)	Independent Parameter(x)	Slope (m)	Intercept (n)
WRS65	Age	-0.39	99.91
WRS65	BC PTA4	-0.52	96.02
WRS65	Vibrogram PTA4	-0.36	96.30
WRS65	FF PTA 4	-0.49	98.70
WRS65	WRSmax unaided	0.39	47.25
WRS65	CE PTA4	-0.05	81.93
WRS65	EG PTA 4	0.40	78.89
WRS65	Dynamic Range PTA4	0.24	69.82

Tab A4 Parameters for the linear fit function referring to the parameters named in Tab A7 and the calculated spearman correlations. Note that the calculated correlation for the effective gain (red-shaded) does not match with the clinical experience. Please see Fig 2 in the manuscript for more details
