# Supplementary material for: Large language model–based prediction of speech intelligibility after Vibrant Soundbridge implantation using multidimensional outcome data: Part 2 of a prospective study
Source: Sci Rep. 2025 Nov 12;15:39564. doi: 10.1038/s41598-025-20919-5 (PMC12612111; doi:10.1038/s41598-025-20919-5)
Supplement: Supplementary file 3 — Supplementary Material 3 [file 41598_2025_20919_MOESM3_ESM.pdf]

Supplemental File 3

Correlation with WRS65 for all 14 predictors

Predictor	r mit WRS65
$WRSmax^2$	0.524
WRSmax	0.52
Vib×WRSmax	-0.198
BC×WRSmax	-0.207
Vib^2	-0.408
Vib	-0.413
BC	-0.431
BC^2	-0.44
BC×Vib	-0.484
age×WRSmax	-0.494
age×BC	-0.64
age×Vib	-0.657
age	-0.663
age^2	-0.665

Tab A5 Correlation with WRS65 for all 14 predictors
