# Supplementary material for: Large language model–based prediction of speech intelligibility after Vibrant Soundbridge implantation using multidimensional outcome data: Part 2 of a prospective study
Source: Sci Rep. 2025 Nov 12;15:39564. doi: 10.1038/s41598-025-20919-5 (PMC12612111; doi:10.1038/s41598-025-20919-5)
Supplement: Supplementary file 4 — Supplementary Material 4 [file 41598_2025_20919_MOESM4_ESM.pdf]

Supplemental File 4 – Expectations and interpretation with regard to the correlation coefficients of the linear regression

✅ 1. Base Predictors

Predictor	Expected Sign	Interpretation
age	negative (↓)	Increased age is associated with reduced speech understanding (WRS65), due to age-related auditory and cognitive decline.
BC	negative (↓)	Higher bone conduction threshold reflects greater sensorineural hearing loss, which reduces WRS65.
Vib	negative (↓)	Indicates coupling efficiency; poorer coupling results in lower speech understanding. Not interpreted as cochlear damage.
WRSmax	positive (↑)	A higher unaided maximum word recognition score predicts better aided performance at 65 dB.

Tab A6 Expected sign of base predictors

🔁 2. Quadratic Terms

Predictor	Expected Sign	Interpretation
age^2	negative (↓)	Accelerated decline in comprehension at higher ages.
BC^2	negative (↓)	Nonlinear worsening of speech understanding with increasing hearing loss.
Vib^2	negative (↓)	Increasing loss in performance with strongly reduced coupling efficiency.
$WRSmax^2$	positive (↑)	Reinforces positive effect; high WRSmax values tend to stabilize at high performance levels.

Tab A7 Expected sign of quadratic terms

🔗 3. Interaction Terms

Interaction	Expected Sign	Interpretation
age × Vib	negative (↓)	Age-related decline interacts with poor coupling efficiency.
age × BC	negative (↓)	Older age amplifies the negative effect of hearing loss.
age × WRSmax	negative (↓)	High WRSmax helps less in older individuals due to central or cognitive limitations.
BC × Vib	negative (↓)	Poor hearing combined with poor coupling creates a compounding negative effect.
BC × WRSmax	negative (↓)	Hearing loss may limit the benefit of good maximum word recognition.
Vib × WRSmax	negative (↓)	Poor coupling may prevent full realization of WRSmax potential.

Tab A8 Expected sign of interaction terms
