# Supplementary material for: Large language model–based prediction of speech intelligibility after Vibrant Soundbridge implantation using multidimensional outcome data: Part 2 of a prospective study
Source: Sci Rep. 2025 Nov 12;15:39564. doi: 10.1038/s41598-025-20919-5 (PMC12612111; doi:10.1038/s41598-025-20919-5)
Supplement: Supplementary file 5 — Supplementary Material 5 [file 41598_2025_20919_MOESM5_ESM.pdf]

Supplemental File 5

Top 50 3-feature models (no sign violations)

Top 10 3-feature models sorted by |sum of coefficients|

Model_ID	features	n_features	R²	r	MAE	RMSE	p-Wert	|sum_coef|
M3X022	a, b, w	3	0.508	0.713	4.668	6.183	0.0086	0.5561
M3X038	a, w, b×w	3	0.506	0.712	4.678	6.195	0.0088	0.505
M3X046	a, w, v×w	3	0.504	0.71	4.695	6.213	0.0092	0.5047999999999999
M3X047	a, w, v²	3	0.504	0.71	4.684	6.212	0.0092	0.4894
M3X009	a, w, b²	3	0.512	0.716	4.629	6.158	0.008	0.47020000000000006
M3X008	a, w, b×v	3	0.512	0.715	4.623	6.161	0.0081	0.4597
M3X006	b, w, a×w	3	0.512	0.716	4.699	6.16	0.0081	0.4484
M3X042	a, w, a×v	3	0.505	0.711	4.694	6.203	0.009	0.4371
M3X049	a, w, a×w	3	0.504	0.71	4.702	6.21	0.0091	0.4339
M3X023	a, w, a×b	3	0.508	0.713	4.718	6.182	0.0085	0.42419999999999997

Tab A9 Top 10 3-feature models sorted by sum of coefficients

Coefficients (rounded, abbreviated)

Model_ID	a	b	v	w	a²	b²	v²	w²	a×w	v×w	b×w	b×v	a×v	a×b
M3X022	-0.2809	-0.0795	nan	0.1957	nan	nan	nan	nan	nan	nan	nan	nan	nan	nan
M3X038	-0.2869	nan	nan	0.2174	nan	nan	nan	nan	nan	nan	-0.0007	nan	nan	nan
M3X046	-0.2961	nan	nan	0.2084	nan	nan	nan	nan	nan	-0.0003	nan	nan	nan	nan
M3X047	-0.2965	nan	nan	0.1925	nan	nan	-0.0004	nan	nan	nan	nan	nan	nan	nan
M3X009	-0.2765	nan	nan	0.1919	nan	-0.0018	nan	nan	nan	nan	nan	nan	nan	nan
M3X008	-0.2762	nan	nan	0.1819	nan	nan	nan	nan	nan	nan	nan	-0.0016	nan	nan
M3X006	nan	-0.0966	nan	0.3486	nan	nan	nan	nan	-0.0032	nan	nan	nan	nan	nan
M3X042	-0.2483	nan	nan	0.1878	nan	nan	nan	nan	nan	nan	nan	nan	-0.0010	nan
M3X049	-0.1275	nan	nan	0.3044	nan	nan	nan	nan	-0.0020	nan	nan	nan	nan	nan
M3X023	-0.2316	nan	nan	0.1909	nan	nan	nan	nan	nan	nan	nan	nan	nan	-0.0017

Tab A10 Top 10 3-feature models: coefficients of linear regression

Top 50 3-feature models (no sign violations)

Top 10 4-feature models sorted by |sum of coefficients|

Model_ID	features	n_features	R^2	r	MAE	RMSE	p-Wert	|sum_coef|
M4V037	a, b, w, a×v	4	0.509	0.713	4.675	6.181	0.0233	0.5216
M4V042	a, b, w, a×b	4	0.509	0.713	4.696	6.181	0.0233	0.4835
M4V013	a, w, b^2, b×v	4	0.513	0.716	4.624	6.157	0.0221	0.4642
M4V046	a, w, v×w, a×b	4	0.508	0.713	4.718	6.182	0.0234	0.4248
M4V047	a, w, v^2, a×b	4	0.508	0.713	4.718	6.182	0.0234	0.4242
M4V019	b, w, a×w, a×v	4	0.512	0.716	4.705	6.158	0.0222	0.421
M4V036	a, w, a×v, a×b	4	0.509	0.713	4.718	6.181	0.0234	0.4119
M4V038	a, w, a×w, b×w	4	0.509	0.713	4.705	6.18	0.0233	0.4072
M4V011	b, w, a^2, a×w	4	0.514	0.717	4.721	6.144	0.0215	0.3633
M4V030	w, a×w, b×w, a×v	4	0.51	0.714	4.717	6.174	0.023	0.3488

Tab A11 Top 10 4-feature models sorted by sum of coefficients

Coefficients (rounded, abbreviated)

Model_ID	a	b	v	w	a^2	b^2	v^2	w^2	a×w	v×w	b×w	b×v	a×v	a×b
M4V037	-0.2618	-0.0699	nan	0.1895	nan	nan	nan	nan	nan	nan	nan	nan	-0.0004	nan
M4V042	-0.2515	-0.0387	nan	0.1923	nan	nan	nan	nan	nan	nan	nan	nan	nan	-0.0010
M4V013	-0.2752	nan	nan	0.1872	nan	-0.0012	nan	nan	nan	nan	nan	-0.0006	nan	nan
M4V046	-0.2319	nan	nan	0.1912	nan	nan	nan	nan	nan	-0.0000	nan	nan	nan	-0.0017
M4V047	-0.2319	nan	nan	0.1906	nan	nan	-0.0000	nan	nan	nan	nan	nan	nan	-0.0017
M4V019	nan	-0.0856	nan	0.3321	nan	nan	nan	nan	-0.0029	nan	nan	nan	-0.0004	nan
M4V036	-0.2230	nan	nan	0.1871	nan	nan	nan	nan	nan	nan	nan	nan	-0.0003	-0.0015
M4V038	-0.0566	nan	nan	0.3472	nan	nan	nan	nan	-0.0026	nan	-0.0008	nan	nan	nan
M4V011	nan	-0.0822	nan	0.2781	-0.0013	nan	nan	nan	-0.0017	nan	nan	nan	nan	nan
M4V030	nan	nan	nan	0.3445	nan	nan	nan	nan	-0.0029	nan	-0.0007	nan	-0.0007	nan

Tab A12 Top 10 4-feature models: coefficients of linear regression

Sigmoidale Fits (L = 100) für ausgewählte Modelle

Modellübersicht

Model_ID	features	x0	k	R^2_sigmoid	r_sigmoid	MAE_sigmoid	RMSE_sigmoid
M3X022	age, BC, WRSmax	57.2941	0.0632	0.502	0.709	4.66	6.22
M4V037	age, BC, WRSmax, age×Vib	57.1941	0.0629	0.502	0.708	4.667	6.225
M4V042	age, BC, WRSmax, age×BC	56.9161	0.0621	0.498	0.706	4.696	6.245
M3X009	age, WRSmax, BC^2	56.6264	0.0613	0.498	0.706	4.657	6.249
M4V013	age, WRSmax, BC^2, BC×Vib	56.6662	0.0614	0.499	0.706	4.647	6.244
M3X008	age, WRSmax, BC×Vib	56.8273	0.0619	0.5	0.707	4.633	6.236

Tab A13 Sigmoidal fit of 6 selected models (3-features and 4-features, three times each)

Vergleich der Regressionsmethoden (Linear, Ridge, Lasso, ElasticNet)

Regressionsvergleich

Model_ID	Regression	Alpha	R²	r	MAE	RMSE
M3X008	ElasticNet	4.3288	0.511	0.715	4.605	6.167
M3X008	Lasso	1.8738	0.511	0.715	4.611	6.164
M3X008	Linear	-	0.512	0.715	4.623	6.161
M3X008	Ridge	756.4633	0.505	0.713	4.585	6.206
M3X009	ElasticNet	3.2745	0.512	0.716	4.619	6.161
M3X009	Lasso	1.2328	0.512	0.716	4.624	6.159
M3X009	Linear	-	0.512	0.716	4.629	6.158
M3X009	Ridge	756.4633	0.505	0.713	4.566	6.202
M3X022	ElasticNet	10.0	0.503	0.712	4.658	6.215
M3X022	Lasso	6.5793	0.501	0.71	4.664	6.228
M3X022	Linear	-	0.508	0.713	4.668	6.183
M3X022	Ridge	1000.0	0.499	0.713	4.611	6.242
M4V013	ElasticNet	5.7224	0.511	0.715	4.6	6.167
M4V013	Lasso	2.4771	0.512	0.716	4.609	6.162
M4V013	Linear	-	0.513	0.716	4.624	6.157
M4V013	Ridge	657.9332	0.507	0.714	4.574	6.193
M4V037	ElasticNet	61.3591	0.432	0.657	5.371	6.644
M4V037	Lasso	30.5386	0.432	0.657	5.371	6.644
M4V037	Linear	-	0.509	0.713	4.675	6.181
M4V037	Ridge	1000.0	0.491	0.702	4.802	6.288
M4V042	ElasticNet	1000.0	0.405	0.64	5.145	6.8
M4V042	Lasso	572.2368	0.404	0.64	5.13	6.808
M4V042	Linear	-	0.509	0.713	4.696	6.181
M4V042	Ridge	1000.0	0.496	0.705	4.776	6.262

Tab A14 Comparison of different regression methods including regularization (LASSO, Rigid regression, Elastic Net) for the 6 chosen models

Modellvergleich: Abweichung und Rang bei negativen Ausreissern

Index	M3X008_Delta	M3X008_Rang	M3X009_Delta	M3X009_Rang	M3X022_Delta	M3X022_Rang	M4V013_Delta	M4V013_Rang	M4V037_Delta	M4V037_Rang	M4V042_Delta	M4V042_Rang
6.00	-8.49	6.00	-7.72	1.00	-7.75	2.00	-8.00	5.00	-7.99	4.00	-7.86	3.00
15.00	-7.44	3.00	-7.53	5.00	-7.32	1.00	-7.53	6.00	-7.40	2.00	-7.45	4.00
17.00	-5.60	3.00	-5.50	1.00	-6.01	6.00	-5.58	2.00	-5.97	5.00	-5.66	4.00
19.00	-15.44	1.00	-15.67	6.00	-15.54	3.00	-15.59	5.00	-15.46	2.00	-15.55	4.00

Tab A15 Comparison between measured values and model predictions across multiple models for four outlier data points (indices 6, 15, 17, 19; deviation from prediction > MAE) from the original dataset. Ranking of models based on prediction accuracy for these outliers.

Modell	Durchschnittlicher Rang
M3X022	3.00
M3X008	3.25
M3X009	3.25
M4V037	3.25
M4V042	3.75
M4V013	4.50

Tab A16 Average ranking across different models.
